# Korean Buddhist Temple Food: A Traditional Plant-Based Dietary Pattern Linking Gut Microbiota, Metabolic Health, and Mindful Living

**DOI:** 10.3390/nu18203331

**Published:** 2026-10-12

**Authors:** Hae-Ji Kang, Young Hoon Jung, Jungwoo Yang, Yun Jeon Lim

**Affiliations:** 1 Department of Microbiology, College of Medicine, Dongguk University, Gyeongju 38066, Republic of Korea; haedi1202@dongguk.ac.kr; 2 Institute of Temple Cuisine and Healing Medicine, College of Medicine, Dongguk University, 123 Dongdae-ro, Gyeongju 38066, Republic of Korea; 3 School of Food Science and Biotechnology, Kyungpook National University, Daegu 41566, Republic of Korea; younghoonjung@knu.ac.kr; 4 Department of Internal Medicine, Dongguk University Ilsan Hospital, Goyang 10326, Republic of Korea

**Keywords:** Korean temple cuisine, fermented foods, short-chain fatty acids, gut microbiome, microbiota-gut–brain axis, Buddhist meditation, vagus nerve, lifestyle medicine

## Abstract

Korean Buddhist temple food represents a traditional plant-based dietary practice rooted in Korean Buddhist monastic traditions and characterized by an emphasis on seasonal and minimally processed plant foods. Traditional temple-food practices generally exclude pungent vegetables—such as garlic, green onion, chives, wild garlic, and asafoetida—and incorporate a variety of plant-based ingredients and fermented foods, such as fermented soybean paste (*doenjang*), soy sauce (*ganjang*), fermented red chili paste (*gochujang*), fermented soybean paste (*cheonggukjang*), and fermented vegetables (*kimchi*). This dietary pattern provides dietary fiber and diverse plant-based phytochemicals, while fermented foods may contain fermentation-associated microorganisms depending on their ingredients, production processes, and storage conditions. These characteristics distinguish Korean Buddhist temple food from dietary patterns dominated by ultra-processed foods (UPFs). This narrative review synthesizes evidence regarding the nutritional characteristics of Korean Buddhist temple food, its potential relevance to gut microbiome ecology and microbial metabolism, and the cultural and contemplative practices associated with its consumption, including ritual communal eating (*baru gongyang*), seated meditation (*chamseon*), and walking meditation (*pohaeng*). Available evidence indicates that several features of temple food, including high intake of plant-based foods, dietary fiber, and fermented foods, provide biologically plausible pathways through which the dietary pattern may influence gut microbial composition and host metabolism. However, direct human evidence linking long-term adherence to Korean Buddhist temple food with gut microbiome composition, microbial function, or microbiota–gut–brain outcomes remains limited. Therefore, the potential health implications of Korean Buddhist temple food should be interpreted in the context of heterogeneous dietary practices and the distinction between direct human evidence and indirect or mechanistic evidence. Further longitudinal and controlled studies are needed to clarify the relationships among temple-food consumption, gut microbiota, microbial metabolites, host physiology, and associated lifestyle practices.

## 1. Introduction

Korean Buddhist temple food (*Sachal Eumsik* in Korean) is a traditional plant-based culinary practice that has evolved over centuries within Korean Buddhist monastic communities [1]. Unlike conventional vegetarian diets, temple food traditionally avoids the five pungent vegetables—garlic, green onion, chives, wild garlic (*Allium monanthum*), and asafoetida (*Ferula assafoetida*)—based on the Buddhist belief that these ingredients may stimulate excessive sensory and emotional activity, thereby interfering with meditative stability and meditative discipline [1,2]. The dietary pattern emphasizes seasonal and minimally processed ingredients, including wild vegetables, mushrooms, seaweeds, legumes, roots, and grains, alongside a wide variety of traditional Korean fermented foods such as *doenjang* (fermented soybean paste), *ganjang* (soy sauce), *gochujang* (fermented red pepper paste), *cheonggukjang* (fast-fermented soybean paste) and *kimchi* (fermented vegetables) [3]. These foods provide dietary fiber, diverse plant-derived phytochemicals, while fermented foods may contain microorganisms associated with the fermentation process, depending on the food matrix, fermentation conditions, processing, and storage [4,5,6]. However, the presence of such microorganisms should not be equated with probiotic status or demonstrated clinical effects. In contrast to dietary patterns characterized by high consumption of ultra-processed foods (UPFs), temple food generally emphasizes whole or minimal processed plant ingredients and traditional preparation methods [2,7]. Nevertheless, the nutritional composition and degree of processing may vary across temples, regions, seasons, and individual dietary practices.

Beyond its nutritional composition, temple food is closely embedded within the broader cultural and contemplative practices of Korean Buddhist monastic life, in which dietary practice is regarded as an integral component of spiritual cultivation rather than merely a means of nourishment [8]. Meals are traditionally practiced through *baru gongyang*, a ritualized and communal form of eating, characterized by silence, gratitude, disciplined awareness, and the use of ceremonial bowls [9]. This practice encourages moderation, sensory awareness, and the minimization of food waste [9]. Although *baru gongyang* shares certain features with contemporary concepts of mindful eating, it is a culturally and religiously embedded practice and should not be considered synonymous with mindful eating without appropriate construct validation. In parallel, contemplative disciplines such as *chamseon* (seated meditation) and *pohaeng* (walking practice) are integrated into daily monastic life, forming a holistic behavioral framework that combines nutrition, mindfulness, meditation, and moderate physical activity [10]. These practices provide an important cultural context for understanding temple-food consumption; however, evidence directly linking specific contemplative practices among Korean Buddhist practitioners to gut microbiome composition, gastrointestinal physiology, or metabolic outcomes remains limited. Findings from meditation, physical-activity, or other behavioral studies may therefore provide mechanistic or indirect context rather than direct evidence for Korean Buddhist temple-food practices [11,12,13,14].

The present narrative review synthesizes current evidence regarding three interrelated dimensions of Korean Buddhist temple food: (1) its nutritional characteristics and emphasis on plant-based and minimally processed food; (2) its potential relevance to gut microbiome modulation through high dietary fiber, fermented food, and other bioactive food components; and (3) the broader cultural and contemplative practices that accompany temple-food consumption and may be relevant to gastrointestinal, metabolic, and psychosocial health. Available evidence suggests that several characteristics of Korean Buddhist temple food provide biologically plausible pathways through which this dietary pattern may interact with gut microbial ecology and host metabolism. However, the strength and directness of evidence vary substantially across outcomes and study populations. Direct human evidence linking long-term adherence to Korean Buddhist temple food with gut microbiome composition, microbial metabolites, or specific host physiological outcomes remains limited. Furthermore, findings from non-Korean monastic contexts (e.g., studies among Thai monks) provide indirect contextual insights into monastic lifestyles but cannot be treated as direct evidence for Korean temple food owing to distinct food compositions and cultural practices. Accordingly, this review distinguishes direct human evidence from indirect and mechanistic evidence and critically considers the extent to which findings from related dietary, microbiome, and contemplative-health research can be applied to Korean Buddhist temple-food practices. The review therefore aims to identify plausible biological pathways and evidence gaps rather than establish causal effects or characterize temple food as an integrated lifestyle medicine model.

## 2. Literature Search and Evidence Selection

Because this article is a narrative review, the literature search was conducted to pro-vide a transparent basis for identifying relevant evidence rather than to perform a systematic review or meta-analysis. Relevant literature was identified through searches of Pub-Med/MEDLINE, Web of Science, and Scopus, supplemented by screening the reference lists of key articles. Search terms combined concepts related to Korean Buddhist temple food and monastic practices (e.g., “Korean temple food,” “Korean Buddhist,” “Buddhist monks,” “Buddhist nuns,” and “temple stay”) with terms related to gut health, nutrition, microbiota, microbial metabolites, fermented foods, meditation, and physical activity. Studies addressing the nutritional characteristics of temple food, gut microbiota and related metabolic or gastrointestinal outcomes, or associated contemplative and lifestyle practices were considered relevant. The identified evidence was classified according to study population, exposure, study design, and evidence directness, distinguishing direct evidence from Korean Buddhist practitioners or clearly defined temple-food exposure from indirect or mechanistic evidence derived from non-monastic, experimental, or related studies. Particular attention was given to differences in dietary composition, ferment-ed-food consumption, seasonality, food preparation, duration of exposure, and monastic versus contemporary temple-stay settings. Evidence was synthesized narratively, with direct human evidence emphasized and indirect or mechanistic findings interpreted as supportive rather than confirmatory evidence.

## 3. Nutritional Characteristics of Korean Temple Food

### 3.1. High Dietary Fiber Content and Abundance of Fermented Foods

Temple food generally emphasizes plant-based ingredients such as whole grains, legumes, vegetables, and seaweeds, which can provide substantial amounts of dietary fiber depending on food selection, preparation, and portion size [15]. Dietary fiber modulates intestinal transit time, augments satiety, and functions as a prebiotic substrate for beneficial colonic microorganisms, thereby promoting the fermentative production of short-chain fatty acids (SCFAs) [16,17]. SCFAs—principally acetate, propionate and butyrate—play pleiotropic roles in host physiology: butyrate serves as the primary energy source for colonocytes, fortifies the intestinal epithelial barrier, and attenuates systemic inflammation via inhibition of histone deacetylases and nuclear factor-κB signaling [17,18,19]. These findings provide a biologically plausible basis for considering dietary fiber as one potential pathway through which plant-rich dietary patterns may influence gut microbial ecology and host physiology; however, the extent to which these mechanisms operate specifically in Korean Buddhist temple-food consumers has not been directly established.

A defining and nutritionally consequential feature of temple food is its extensive incorporation of traditional fermented foods [2]. *Doenjang*, *ganjang*, *gochujang*, *cheonggukjang*, and *kimchi* may collectively constitute a rich source of viable lactic acid bacteria (LAB; mainly *Lactobacillus* spp. and *Leuconostoc* spp.), yeasts, and their bioactive metabolites [20,21]. The fermentation process enhances the bioavailability of micronutrients, degrades antinutritional factors such as phytates and trypsin inhibitors, and directly delivers fermentation-derived microorganisms to the gastrointestinal tract [4,22]. The presence of viable microorganisms in fermented foods should not, however, be equated with probiotic status, which requires specific strain-level characterization and evidence of a health benefit. Similarly, the co-occurrence of dietary fiber and fermented foods does not by itself establish a “synbiotic” dietary pattern. Therefore, fermented foods in Korean Buddhist temple food are best considered potential contributors to dietary microbial exposure and food-derived bioactive compounds, rather than established probiotic or synbiotic interventions [23].

### 3.2. Abundance of Antioxidant Phytonutrients

The seasonal wild greens and diverse vegetables central to temple food are rich sources of antioxidant phytonutrients, including carotenoids such as β-carotene and lutein, as well as polyphenols and flavonoids, together with vitamins C and E [24,25,26,27]. β-Carotene undergoes enzymatic cleavage to retinol in the intestinal mucosa, supporting vision, immune competence, and epithelial integrity, while simultaneously quenching reactive oxygen species and mitigating oxidative stress [24,28]. Fermented soybean products (i.e., *doenjang* and *cheonggukjang*) are particularly enriched in isoflavones—notably genistein and daidzein—that exhibit estrogen-receptor-modulating activity alongside potent antioxidant and anti-inflammatory properties [26,29,30]. These findings provide a plausible basis for considering the diverse phytochemical composition of temple food as a potential contributor to its biological effects; however, the presence of individual bioactive compounds does not establish specific health benefits of the overall temple-food dietary pattern. The concept of “food synergy” suggests that interactions among nutrients and bioactive compounds within whole foods or dietary patterns may contribute to biological effects beyond those of individual components, but such interactions have not been directly established in Korean Buddhist temple-food consumers [31].

### 3.3. Restriction of Animal Protein and Emphasis on Seasonal Whole Ingredients

Temple food categorically excludes animal-derived foods, including meat, poultry, fish, and eggs, relying instead on plant-based protein sources such as tofu, legumes, and nuts [2]. Dietary analyses of vegetarian populations have broadly reported lower intake of saturated fat and limited consumption of heme iron, alongside greater contributions of plant-derived foods and non-heme iron [32]. Such dietary characteristics might contribute to more favorable cardiometabolic profiles, including lower circulating low-density lipoprotein (LDL) cholesterol and reduced concentrations of trimethylamine N-oxide (TMAO), a gut microbiota-associated metabolite linked to cardiovascular risk [33,34]. However, findings from vegetarian populations cannot be assumed to represent the specific effects of Korean Buddhist temple food, given differences in dietary composition, food preparation, duration of adherence, and other lifestyle factors. The plant-rich composition of temple food also provides dietary fiber and diverse phytochemicals that may influence microbial fermentation and gut microbial ecology [35]. These features provide biologically plausible pathways through which a plant-based dietary pattern may affect gut and metabolic health, but direct evidence in Korean Buddhist temple-food consumers remains limited.

Another distinctive feature of temple food is its emphasis on seasonal and fresh whole ingredients [1,2]. Seasonal vegetables, wild greens, mushrooms, legumes, and grains are selected according to their availability and freshness, potentially contributing to dietary variety and exposure to diverse nutrients and plant-derived bioactive compounds [36]. However, the specific composition and diversity of ingredients may vary according to season, region, temple, and individual dietary practices, and the health implications of this seasonal dietary pattern have not been directly established.

### 3.4. Temple Food as a Minimally Processed Diet

The NOVA classification system categorizes foods into four groups based on the extent and purpose of industrial processing [37,38]. Within this framework, ultra-processed foods (UPFs) are defined as industrial formulations composed predominantly of chemically modified food substances and additives, containing little to no whole food content [37,38]. Accumulating evidence suggests that high UPF consumption may be associated with adverse metabolic and microbial outcomes, while emerging research has also examined potential associations with alterations in gut microbial composition and function [39,40]. In contrast, Korean Buddhist temple food generally emphasizes whole or minimally processed plant ingredients and traditional food-preparation methods, although individual foods and preparation practices may involve different degrees and types of processing. Therefore, temple food should not be considered uniformly equivalent to a specific NOVA category, and its classification may vary according to the individual food and preparation method. Many temple-food ingredients and preparations would likely fall within NOVA Group 1 (unprocessed or minimally processed foods) and Group 3 (processed foods prepared via traditional methods), while traditionally emphasizing the use of whole ingredients and limiting the use of industrial additives [37,38]. This distinct dietary profile positions temple food as a compelling model for investigating how minimally processed, plant-based diets can counteract the metabolic and microbial perturbations associated with modern Western diets [35,41]. However, whether these differences translate into specific metabolic or gut microbiome benefits among Korean Buddhist temple-food consumers remains insufficiently established.

## 4. Korean Temple Food and Gut Microbiome Modulation

The gut microbiome—comprising trillions of microorganisms residing principally in the colon—maintains bidirectional interactions with immune, metabolic, endocrine, and neurological systems [42]. Diet is widely regarded as the most potent modifiable determinant of gut microbiome composition [43]. Two key features of temple food—high dietary fiber intake and regular consumption of fermented foods—exert complementary yet mechanistically distinct effects on gut microbial ecology, as discussed below [44,45].

### 4.1. High-Fiber Diet, Gut Microbiome, and Glucagon-like Peptide-1 (GLP-1)

Dietary fiber resists digestion by host enzymes and arrives intact in the colon, serving as a major substrate influencing gut microbial ecology and host metabolic signaling [44,46,47,48]. Colonic fermentation of these complex carbohydrates can promote the abundance of various saccharolytic bacteria, potentially including taxa such as *Bifidobacterium* spp., *Lactobacillus* spp., *Faecalibacterium prausnitzii*, and *Akkermansia muciniphila*, depending on fiber structure, dose, and baseline host microbiome composition [47,48]. Among these, *F*. *prausnitzii* functions as a pivotal anti-inflammatory commensal whose abundance correlates inversely with inflammatory bowel disease severity in observational studies, while *A*. *muciniphila* reinforces the intestinal mucus layer and is associated with improved insulin sensitivity [47,49,50]. Together with microbiota-derived metabolites, including short-chain fatty acids (SCFAs) and polyphenol-derived metabolites (e.g., urolithin A), these microbial functions may contribute to the maintenance of host metabolic health [51,52,53]. Evidence from murine models demonstrates that dietary fiber plays a structural role in preserving colonic mucus and microbial architecture [54,55]. The importance of sustained fiber intake is further underscored by evidence that dietary deprivation can progressively reshape the gut microbial ecosystem [56]. Furthermore, experimental studies indicate that prolonged dietary fiber deprivation can progressively alter gut microbiome ecology and lead to persistent loss of microbial taxa, including depletion of key taxa such as *Prevotella* spp. [56,57]. In contrast, high-fiber dietary intervention in humans and experimental models can rapidly reshape the gut microbiota [58]. Consequently, this shift promotes SCFA production, thereby acidifying the luminal environment, which suppresses pathobiont expansion [59,60]. Importantly, these microbial metabolites provide a mechanistic link between dietary fiber, intestinal ecology, and systemic metabolic regulation [61,62,63]. In cell-based and animal models, as well as select clinical studies, SCFAs serve as endogenous ligands for free fatty acid receptors 2 and 3 (FFAR2/FFAR3) on enteroendocrine L-cells, triggering the secretion of glucagon-like peptide-1 (GLP-1) to enhance glucose-stimulated insulin release, suppress glucagon, and promote satiety [64,65,66]. Concurrently, butyrate promotes colonic regulatory T cell (Treg) differentiation, thereby maintaining gut immune tolerance [67,68]. However, these pathways should not be interpreted as a single linear fiber–microbiome–SCFA–GLP-1–Treg axis, and their relative contributions may depend on dietary composition, microbial ecology, host physiology, and experimental context. Importantly, although these mechanisms provide a biologically plausible framework for understanding how fiber-rich dietary patterns may influence gut and metabolic health, they have not been directly demonstrated in Korean Buddhist temple-food consumers.

### 4.2. Fermented Food Consumption and Gut Microbiome Changes

In a randomized controlled dietary intervention study, a 10-week diet enriched in fermented foods increased gut microbiome diversity and reduced circulating inflammatory markers compared to a high-fiber diet [69]. However, subsequent research has emphasized that these effects are not uniform across all fermented foods and instead depend on the food matrix, fermentation process, microbial composition, dose, and individual host microbiome characteristics [70,71,72]. Collectively, recent evidence further emphasizes that fermented foods can influence the gut microbiome through interactions involving food-associated microorganisms, fermentation-derived metabolites, and the resident microbial community [73,74,75]. The relative contribution of these components to gut microbial and host responses remains context-dependent and has not been established uniformly across fermented foods.

Korean Buddhist temple food commonly incorporates a variety of traditionally fermented foods, including *jang* products such as *ganjang*, *doenjang*, and *gochujang*, as well as fermented vegetables such as *kimchi*, with ingredients and preparation practices varying across temples and regions. These foods may contain fermentation-associated microorganisms, including lactic acid bacteria and, depending on the food and fermentation process, other microorganisms such as *Bacillus* spp., together with fermentation-derived metabolites and other bioactive compounds. However, the microbial composition and viability of these foods can vary substantially according to ingredients, fermentation conditions, maturation, storage, and subsequent processing. Accordingly, Korean temple food should not be considered a standardized source of live microorganisms or a defined probiotic intervention [3,5,6,20]. Unlike industrially processed fermented products, fermented soybean foods such as *doenjang* and *ganjang* contain diverse fermentation-derived metabolites and bioactive compounds, including peptides and polyphenol-related compounds [76,77]. The regular consumption of fermented foods within a plant-rich temple-food pattern therefore represents a biologically plausible source of dietary microbial and metabolite exposure, but direct evidence demonstrating that temple-food consumption increases gut microbial diversity or reduces systemic inflammation in Korean Buddhist practitioners remains limited.

## 5. Temple Food and Associated Lifestyle Practices

The health-promoting potential of temple food cannot be fully appreciated when isolation from the contemplative lifestyle in which it is embedded. Korean Buddhist monastic culture integrates dietary practice with *baru gongyang* (ritualized mindful eating), *chamseon* (seated meditation), and *pohaeng* (meditative walking)—a combination of lifestyle practices that may synergistically modulate stress physiology, autonomic nervous system balance, and physical activity levels through mechanisms that independently enhance gut microbiome health [1,9,10,14].

### 5.1. Baru Gongyang: Restraint and Gratitude as the Framework for Mindful Eating

*Baru gongyang* is a monastic ritual meal in which practitioners eat from four nested wooden bowls (baru: rice bowl, soup bowl, side-dish bowl, and water bowl), serve themselves only what they can consume, and finally rinse each bowl with water which is then consumed—leaving no residue [8,9]. The practice encapsulates two cardinal Buddhist values: restraint (*jeolche*, operationalized as portion-controlled eating commensurate with physiological need, and gratitude (*gamsa*, cultivated through silent contemplation of the causal chain that produced the meal) [8,9]. Although these features share certain characteristics with contemporary concepts of mindful eating, *baru gongyang* is a culturally and religiously embedded Buddhist practice and should not be considered conceptually equivalent to mindful eating without appropriate construct validation [10,78].

Mindful eating interventions have been associated with improvements in maladaptive eating behaviors, greater awareness of internal hunger and satiety cues, and a more adaptive psychological relationship with food [14,79,80]. These findings may provide relevant contextual evidence for understanding some behavioral features of *baru gongyang*, but they do not establish that participation in *baru gongyang* produces comparable effects. Beyond individual eating behavior, the communal practice of *baru gongyang* may provide an additional psychosocial pathway by fostering social connectedness and interpersonal bonding. Indeed, shared meals have been linked to greater perceived social connectedness, trust, and life satisfaction [81,82]. More broadly, socially supportive relationships and a sense of group belonging have been shown to buffer neuroendocrine stress reactivity, including hypothalamic–pituitary–adrenal (HPA) axis activity, although this buffering effect has been demonstrated mainly for social support and group identity in general rather than for commensal eating specifically [83,84]. Psychological stress, partly through HPA-axis activation, has systemic effects on immune, cardiovascular, and metabolic function, and may similarly influence the gut microenvironment through neuroendocrine and immune pathways [85,86]. However, these findings derive primarily from studies of social support, stress, or mindful-eating interventions rather than from studies directly evaluating *baru gongyang*. Therefore, they should be regarded as indirect or contextual evidence rather than evidence demonstrating specific physiological or gut-related effects of the practice.

Taken together, the contemplative and communal features of *baru gongyang* provide a plausible context in which eating behavior, social interaction, and stress-related processes may intersect [87,88,89]. Nevertheless, the proposed relationships among *baru gongyang*, mindful eating, stress regulation, and gut–brain health remain hypothetical and have not been directly established in Korean Buddhist practitioners. Future studies should therefore assess these constructs within the cultural and religious context of baru gongyang, using validated behavioral, physiological, and, where appropriate, gut microbiome-related measures.

### 5.2. Chamseon and Pohaeng: Integrating Meditation and Physical Activity

*Chamseon* (seated Zen meditation) constitutes a central contemplative practice within the Korean Buddhist tradition [10]. Neuroimaging and psychophysiological studies reported that regular meditation is associated with functional changes in prefrontal and limbic brain regions, including enhanced prefrontal regulatory activity and attenuated amygdala reactivity to stress-related stimuli [90,91]. These adaptations have been associated with changes in stress appraisal and physiological stress responses in mindfulness-related research [90,91,92,93]. Meditation has been associated with changes in autonomic and physiological stress regulation [90,91,92,93]. The vagus nerve represents an important bidirectional pathway within the gut–brain axis, integrating signals between the gastrointestinal tract, brainstem, autonomic nervous system, and higher-order brain regions [94,95]. Consequently, persistent psychological stress and impaired autonomic regulation can adversely affect gastrointestinal function, intestinal barrier integrity, visceral sensitivity, and the gut microbial ecosystem [94,95,96]. However, these findings are derived primarily from meditation or stress-related studies conducted outside Korean Buddhist monastic settings and therefore provide indirect or contextual evidence rather than direct evidence for the physiological or gut-related effects of *chamseon*.

*Pohaeng*, a form of walking meditation traditionally practiced between periods of seated meditation, involves slow, deliberate walking with sustained attention to bodily movement and each footfall [97]. It thus integrates low-intensity physical activity with sustained attentional engagement. Regular physical activity and exercise have been associated with changes in gastrointestinal motility and function, although findings vary according to exercise characteristics and individual factors [98,99,100]. Beyond these gastrointestinal effects, habitual physical activity has been associated with increased gut microbiome diversity and enrichment of short-chain fatty acid (SCFA)-producing taxa, particularly butyrate-producing organisms such as *Roseburia* spp. (e.g., *R*. *intestinalis*), *Faecalibacterium prausnitzii*, and members of the *Lachnospiraceae* family [101,102,103]. However, the magnitude and direction of these associations vary according to exercise intensity, duration, diet, host characteristics, and baseline microbiome composition [104,105]. findings from physical-activity research may provide biological context for considering pohaeng but should not be interpreted as direct evidence of its effects in Korean Buddhist practitioners.

Taken together, *chamseon* and *pohaeng* represent culturally embedded contemplative and behavioral practices that may involve psychological regulation and physical activity, respectively. Their potential relevance to gastrointestinal, metabolic, or gut–brain health remains biologically plausible but insufficiently characterized. Further studies conducted in Korean Buddhist practitioners are needed to determine whether these practices are associated with measurable changes in stress-related physiology, gastrointestinal function, gut microbiota, or related metabolic outcomes.

## 6. Limitations and Future Directions

Several limitations should be considered when interpreting the evidence reviewed in this review. First, as a narrative review, the available evidence is heterogeneous in terms of study design, population, dietary exposure, and outcome measures. Direct human evidence linking long-term Korean Buddhist temple-food consumption with gut microbiota composition, microbial metabolites, gastrointestinal function, or metabolic outcomes remains limited. Much of the available evidence is derived from related dietary, microbiome, meditation, physical-activity, or experimental studies and therefore provides indirect or mechanistic rather than direct evidence. In addition, temple-food practices vary according to temple, region, season, food preparation, and duration of adherence, while co-occurring lifestyle factors may confound observed associations.

Importantly, the potential health benefits of temple food should be considered alongside its overall nutritional adequacy and food quality. A plant-rich dietary pattern can provide substantial dietary fiber and diverse phytochemicals, while thoughtful meal planning can help ensure adequate protein quantity and quality, as well as sufficient vitamin B12, iron, zinc, calcium, vitamin D, and omega-3 fatty acids, particularly when animal-source foods are largely or entirely excluded [106,107]. Fermented foods commonly used in temple cuisine, such as *doenjang*, *ganjang*, *gochujang*, and *kimchi*, can contribute to sodium intake, although their contribution varies with the recipe, portion size, and frequency of consumption [108]. Moderation and lower-sodium preparation methods may therefore help support a balanced dietary pattern. As with other traditionally fermented foods, appropriate processing and storage practices are important for maintaining quality and minimizing potential food-safety concerns. Some fermented foods may also contain viable microorganisms; however, their presence alone does not establish probiotic benefits, which require evidence for specific strains and intended uses. Their suitability may warrant individual consideration in people with particular health conditions, including those with compromised immune function.

Future studies should therefore prioritize well-characterized longitudinal and controlled studies involving Korean Buddhist practitioners and clearly defined temple-food exposures. Dietary composition, fermented-food type and quantity, sodium intake, nutrient adequacy, duration and adherence, and appropriate comparison groups should be systematically assessed. Future research should integrate nutritional and clinical outcomes with gut microbiome profiling, microbial metabolites such as short-chain fatty acids, gastrointestinal and metabolic biomarkers, and validated measures of contemplative and behavioral practices. Such studies would help distinguish the effects of temple-food consumption from those of associated monastic lifestyle factors and determine whether the proposed nutritional, microbiome-related, and physiological mechanisms are supported by direct human evidence. Balanced assessment of both potential benefits and nutritional or safety risks will be essential for determining the health implications of Korean Buddhist temple food.

## 7. Conclusions

Korean Buddhist temple food is a culturally distinctive plant-based dietary pattern emphasizing seasonal, minimally processed plant foods, dietary fiber, and traditional fermented foods. These features suggest plausible links to gut microbial ecology, microbial metabolism, and host health. Associated practices, including *baru gongyang*, *chamseon*, and *pohaeng*, may also be relevant to dietary behavior and stress regulation. However, direct human evidence for the health effects of this combined dietary and lifestyle pattern remains limited, as most supporting findings derive from studies of individual components or settings outside Korean Buddhist monastic life. Korean Buddhist temple food may therefore be considered a traditional dietary pattern with potential nutritional and microbiome-related relevance, while evidence supporting its use as a health intervention remains limited. Well-characterized longitudinal and controlled studies are needed to clarify how dietary composition, fermented-food intake, long-term adherence, and associated practices relate to microbial, nutritional, gastrointestinal, metabolic, and psychological outcomes. Such research would help distinguish biologically plausible hypotheses from associations supported by direct human evidence.

## Data Availability

No new data were created or analyzed in this study. Data sharing is not applicable to this article.

## Abbreviations

The following abbreviations are used in this manuscript:

GBA	gut–brain axis
FFAR2	free fatty acid receptor 2
FFAR3	free fatty acid receptor 3
GLP-1	glucagon-like peptide-1
GPR41	G protein-coupled receptor 41
GPR43	G protein-coupled receptor 43
HPA	hypothalamic–pituitary–adrenal
LAB	lactic acid bacteria
LDL	low-density lipoprotein
SCFAs	short-chain fatty acids
TMAO	trimethylamine N-oxide
Treg	regulatory T cell
UPFs	ultra-processed foods
